# Mental health and coping strategies in graduate students in the COVID-19 pandemic

**DOI:** 10.1590/1518-8345.5012.3491

**Published:** 2021-10-29

**Authors:** Fabio Scorsolini-Comin, Naiana Dapieve Patias, Alisson Junior Cozzer, Pedro Augusto Warken Flores, Jean Von Hohendorff

**Affiliations:** 1 Universidade de São Paulo, Escola de Enfermagem de Ribeirão Preto, PAHO/WHO Collaborating Centre for Nursing Research Development, Ribeirão Preto, SP, Brazil.; 2 Scholarship holder at the Conselho Nacional de Desenvolvimento Científico e Tecnológico (CNPq), Brazil.; 3 Universidade Federal de Santa Maria, Centro de Ciências Sociais e Humanas, Santa Maria, RS, Brazil.; 4 Faculdade Meridional, Escola de Saúde, Passo Fundo, RS, Brazil.

**Keywords:** Psychological Adaptation, COVID-19, Depression, Anxiety, Psychological Stress, Student’s Health, Adaptación Psicológica, COVID-19, Depresión, Ansiedad, Estrés Psicológico, Salud del Estudiante, Adaptação Psicológica, COVID-19, Depressão, Ansiedade, Estresse Psicológico, Saúde do Estudante

## Abstract

**Objective:**

to verify the relation of depression, anxiety, and stress symptoms with coping strategies in graduate students in the context of the new coronavirus pandemic (COVID-19).

**Method:**

an electronic cross-sectional and correlational survey was conducted with 331 Brazilian graduate students, aged 20-64 years old, who answered an online form containing a sociodemographic data questionnaire, a coping strategies scale, and the DASS-21 scale. Descriptive analysis, Mann-Whitney U or Kruskal-Wallis tests, and Spearman’s correlation were performed.

**Results:**

the main results indicated that maintaining work and study routines, as well as a religious practice, is correlated with lower scores of depression, anxiety, and stress symptoms, as well as with coping strategies that can act as protective factors.

**Conclusion:**

the new coronavirus pandemic has strained public health and increased the need for studies aimed at understanding the impact of the event on the mental health of the population. It is suggested that employment and religiousness should be considered in interventions with graduate students.

## Abstract (es)

**Objetivo:**

verificar la relación de los síntomas de depresión, ansiedad y estrés con las estrategias de afrontamiento en estudiantes de postgrado en el contexto de la pandemia ocasionada por el nuevo coronavirus (COVID-19).

**Método:**

se realizó un estudio electrónico de investigación transversal y correlacional con 331 estudiantes de postgrado brasileños de 20 a 64 años de edad, que respondieron un formulario en línea que incluía un cuestionario sobre datos sociodemográficos, una escala de estrategias de afrontamiento y la escala DASS-21. Se realizó un análisis descriptivo, al igual que las pruebas U de Mann-Whitney, de Kruskal-Wallis y de correlación de Spearman.

**Resultados:**

los resultados principales indicaron que mantener rutinas de trabajo y estudio, al igual que profesar una religión, están correlacionados con puntajes más bajos en términos de síntomas de depresión, ansiedad y estrés, al igual que con estrategias de afrontamiento que pueden servir como factores de protección.

**Conclusión:**

la pandemia ocasionada por el nuevo coronavirus ha sobrecargado a la salud pública e incrementado la necesidad de estudios dirigidos a comprender el efecto del fenómeno sobre la salud mental de la población. Se sugiere que tener empleo y profesar una religión deberían ser factores a considerar en intervenciones con estudiantes de postgrado.

## Abstract (pt)

**Objetivo:**

verificar a relação entre sintomas de depressão, ansiedade, estresse e as estratégias de *coping* em pós-graduandos no contexto da pandemia do novo coronavírus (COVID-19).

**Método:**

pesquisa eletrônica transversal e correlacional conduzida com 331 estudantes de pós-graduação brasileiros, com idades entre 20 e 64 anos que responderam a um formulário online contendo um questionário de dados sociodemográficos, uma escala de estratégias de *coping* e a escala DASS-21. Foram realizadas análises descritivas, análises U de Mann-Whitney ou Kruskal-Wallis e análises de correlação Spearman.

**Resultados:**

os principais resultados indicaram que a manutenção de uma rotina laboral e de estudos, bem como a prática religiosa estão correlacionadas a menores escores de sintomas de depressão, ansiedade e estresse, bem como com estratégias de enfrentamento que podem funcionar como fatores protetivos.

**Conclusão:**

a pandemia do novo coronavírus prejudicou a saúde pública e aumentou a necessidade de estudos voltados para a compreensão do impacto desse evento na saúde mental da população. Sugere-se que a inserção no mercado de trabalho e a religiosidade sejam consideradas em intervenções com pós-graduandos.

## Introduction

In relation to mental health, the population of university students is being more researched, representing an increasing trend since the COVID-19 pandemic^(1-4)^. The expressive levels of psychopathological symptoms found in the contemporary world are not the only scientific interest in these students. The way in which Higher Education Institutions (HEIs) have been organized is questioned, considering hospitality to the students as one of the guidelines to offer more humanized academic education, in an attempt to go beyond the mere focus on the cognitive aspects and practical training of future professionals, allowing the construction of strategies for higher education quality, in which the emotional conditions are taken into account for this academic training to be successful^(5)^.

Research in mental health in Brazilian and foreign universities has prioritized undergraduate students, with a reduced number of studies depicting the reality of graduate students, causing certain invisibility of this population^(6)^. Mental health in graduate students has been an urgent issue, although less researched. In recent years, graduate programs have undergone important changes regarding their organization, incentives for the growing production of innovative knowledge, and communication possibilities with research studies and researchers from abroad, expanding collaboration networks and, consequently, the scientific and social impact of this production^(7)^.

Despite these challenges, which consider the need to innovate and internationalize graduate education, the mental health of these students, often neglected by graduate programs, should be discussed. Elements that must be problematized in this discussion are the study, work and permanence at the University conditions throughout graduate education. This discussion must consider the socio-economic conditions of these graduates, the instability of graduate programs in the Brazilian political scenario, and the insertion into the job market after the pandemic. Such combined elements should be compared to an expanded approach to mental health in graduate education, retrieving the need to explore the strategies by the students to face these markers, and unequivocally leading us to the concept of coping^(1,8)^.

Coping can be understood as the association of strategies that people use as a way to eliminate, reduce or manage stressful events. Such strategies can be focused on the problem or on emotion; the former emphasizing the cause of stress and changing its relation with it, and the latter focusing on the emotional response and adaptation to the event^(8)^. Thus, one of the resulting needs refers to how graduate students have adapted to the challenges imposed by the COVID-19 pandemic, with resources and strategies not only to maintain teaching and research activities, but also for mental health in this period of strong emotional distress.

This interest reveals the need not only to know the psychopathological symptoms of this group, but their conditions to face the challenges imposed. A number of studies conducted with different student populations have highlighted resources such as mood, distraction, and also resilience capability^(9-12)^. These strategies are focused on both emotional aspects and problem solving. However, the associations between the production of these resources and the emotional conditions of graduate students are still unclear in the scientific literature^(3,10,12)^.

Cultural contexts can exert an impact on these strategies, as in the case of Brazilian students^(10)^. Knowing these scenarios can make educational and mental health strategies aimed at these students even more effective in a context of transit through the pandemic, which reinforces the relevance of this research. The interest in this study arose from the need to know if the social distancing policies, launched in Brazil from March 2020 and imposed during the pandemic, had repercussions on the mental health of these students.

From this panorama, this study focuses on possible coping strategies produced in response to COVID-19 and on the recognition of the psychological needs of graduate students in the pandemic. Thus, it aims at verifying the relation of depression, anxiety, and stress symptoms with coping strategies in graduate students in the context of the new coronavirus pandemic (COVID-19).

## Method

### Study design

An electronic cross-sectional and correlational survey was conducted.

### Scope

Students enrolled in graduate courses during the first semester of 2020 were eligible to participate in this study. These students should be enrolled and at any stage of their graduate studies, without restrictions on the educational institution or training or research area. The study included 331 Brazilian graduate students. Sample size was calculated using the OpenEpi software, version 3.01, considering a confidence level of 95%, a margin of sampling error of 5% and a population of 288,538 graduate students^(13)^.

### Data-collection instruments

A sociodemographic data sheet, specifically designed for this study with questions related to the characterization of the participants, such as gender, age, schooling level and information on the degree of social distancing, COVID-19 symptoms, testing and results, as well as socioeconomic, labor and educational situation.

Coping Strategies Inventory (CSI). The Brazilian version^(8)^ consists of 66 items, divided into 8 factors: Factor 1 - Confrontation; Factor 2 - Distancing; Factor 3 - Self-control; Factor 4 - Social Support; Factor 5 - Accepting Responsibility; Factor 6 - Escape-avoidance; Factor 7 - Problem Solving; and Factor 8 - Positive Reappraisal. The respondents must indicate what they did in a given situation according to the classification, ranging from 0- strategy not used; 1- used somewhat; 2- used quite a bit; to 3- used a great deal. In this study, the situation was described as “social distancing due to COVID-19” and the overall Cronbach’s Alpha was 0.90.

The Depression, Anxiety and Stress Scale (DASS-21) - Short Form, validated for the Brazilian adult population^(14)^. It is based on the tripartite model, which divides the anxiety and depression symptoms into three basic structures. In DASS-21, the participants indicate the degree to which they experienced each of the symptoms described during the last week (previous week), on a 4-point Likert scale ranging from 0 (Did not apply to me at all) to 3 (Applied to me to a considerable degree or a good part of time). The scores for depression, anxiety and stress are determined by the sum of the scores of each of the 21 items. In this study, the overall Cronbach’s alpha was 0.95: 0.95 for depression, 0.88 for anxiety, and 0.90 for stress, which can be considered good psychometric indexes. Symptom severity in DASS-21 is calculated by multiplying the mean scores by two, the results being: normal, mild, moderate, severe, and extremely severe.

### Data collection

Online data collection using the Google Forms tool. The invitation containing the link to the form was sent via e-mail to several Brazilian Higher Education Institutions. Invitations were also sent to groups of graduate students via Facebook, and individually via WhatsApp. The researchers also created a profile for the research on Instagram, where the survey link remained available. The invitations contained a brief survey description and a link to answer the online form. Collection took place between May10^th^ and June20^th^,2020. The participants accepted to take part in the research through the Free and Informed Consent Form(FICF), which was made available in text format on the first page, before the data collection instruments.

### Data analysis

Initially, normality tests (Kolmogorov-Smirnov and Shapiro-Wilk) were performed, which showed that the data regarding the depression, anxiety and stress symptoms and coping strategies variables did not present normal distribution (p < 0.05) and, therefore, non-parametric statistics should be employed. Subsequently, descriptive analysis (frequency, mean, standard deviation) were performed to investigate the graduate students’ profiles and the characteristics related to social distancing and COVID-19. Afterward, the Mann-Whitney U test was performed to verify differences in depression, anxiety and stress symptoms and coping strategies employed by group (gender, religious practice and employment situation). The Kruskal-Wallis test was performed to verify the same symptoms by type of institution (private, public, community), study situation and type of social distancing. To verify if there was any correlation between the depression, anxiety and stress symptoms and the coping strategies employed, Spearman’s correlation analyses were performed.

Finally, effect size tests were performed in groups where there were statistically significant differences in the constructs under study. The effect size test (r) was performed by calculating r=Z/√N (in pairs), using the following values as parameters: small (0.2), medium (0.5), and large (>0.8)^(15)^. The correlations were classified according to the following dimensions of magnitude: weak (<0.3), moderate (0.3 to 0.59), strong (0.60 to 0.99), or perfect (1.0)^(16)^.

### Ethical aspects

Ethical approval was obtained from the Institutional Review Board of the Federal University of Santa Maria (Opinion Number: 4,022,969).

## Results

The study included 331 graduates, aged 20-64 years old (M = 31.42; SD = 7.66), 75% (n = 248) female, 73% (n = 242) white-skinned, 75% (n = 249) from public universities, 58% (n = 193) pursuing a master’s degree, 48% (n = 160) in the area of Human Sciences, 43% (n = 144) with a family income of 2 to 6 minimum wages, and 51% (n = 167) practicing some type of religion. Regarding their employment situation, 55% (n = 183) have a job, of which 19% (n = 62) need to go out of their house to work (Table 1).

**Table 1:** Sociodemographic characteristics. Brazil (n = 331)

Characteristics	n	%	Characteristics	n	%
State			Study situation		
RS	121	37	Yes	209	63
SP	29	9	Partially	101	31
MG	26	8	No	21	6
SC	22	7	Institution		
Others	133	270	Public	249	75
Gender			Private	71	22
Female	248	75	Community	11	3
Male	80	24	Skin color		
Rather not say	3	1	White	242	73
Graduate studies			Brown	62	19
Master’s degree	193	58	Black	24	7
Doctorate degree	131	40	Asian	3	1
Did not answer	7	2	Works		
Field of study			Yes	183	55
Humanities	160	48	No	133	40
Health Sciences	56	17	Temporarily suspended	15	5
Applied Social Science	38	12	Do you currently need to go out of your house to work?		
Engineering	23	7	No	232	70
Others	54	16	Yes	62	19
Religious practice			Did not answer	37	11
Yes	167	51	Family income		
No	164	49	Up to 2 minimum wages*	48	15
Religion			2 to 4 minimum wages*	73	22
Catholicism	83	25	4 to 6 minimum wages*	71	22
Spiritism	34	10	6 to 8 minimum wages*	56	17
Mission Protestantism	27	8	8 to 10 minimum wages*	30	9
Protestantism of Pentecostal origin	11	3	10 minimum wages* or more	53	16

* Current minimum wage: R$ 1,045.00. Brazil, 2020

In terms of sample characterization, when asked about the characteristics related to social distancing and COVID-19, 3% (n = 11) indicated that they had symptoms and 4% (n = 14) answered that they had been tested, with a positive result in 8% (n = 2) of the cases. Concerning housing arrangement during the pandemic, 54% (n = 176) live with two to three people and, of them, 47% (n = 156) are at risk. In addition, most of the respondents reported being in partial social distancing, going out of their house once a week (40%, n = 133) only to buy essential products. These data are summarized in Table 2.

**Table 2:** Characteristics related to COVID-19. Brazil

Characteristics	n	%	Characteristics	n	%
COVID-19 symptoms			Social distancing degree		
Yes	11	3	Partial	277	84
No	320	97	Total	42	13
COVID-19 test performed			None	12	3
Yes	14	4	How many days do you go out of your house in a given week?		
No	317	96	None	46	14
Test result			1	133	40
Negative	12	86	2	59	18
Positive	2	14	3	38	12
Extra residents			4	19	6
None, I live alone	22	7	5	18	5
1	54	16	6	13	4
2	108	33	7	5	1
3	68	21	At-risk group people		
4	55	17	Yes	156	47
5	18	5	No	138	42
6	6	1	I live alone	37	11

Regarding psychopathological symptoms, graduate students had their scores of depression (M = 15.28; SD = 6.21), anxiety (M = 5.93; SD = 5.66) and stress (M=10.37; SD=5.88) classified as moderate. Regarding the coping strategies used, the highest means were in the positive reappraisal strategy (M = 9.80, SD = 4.76) and the lowest were found in accepting responsibility (M = 4.27, SD = 2.64) (Table 3).

**Table 3:** Descriptive analysis of depression, anxiety and stress symptoms and coping strategies. Brazil

	M*	SD^†^
Depression	7.64	6.21
Anxiety	5.93	5.66
Stress	10.37	5.88
CSI^‡^1 - Confrontation	5.46	2.79
CSI^‡^2 - Distancing	6.63	3.10
CSI^‡^3 - Self-control	9.58	3.37
CSI^‡^4 - Social Support	9.26	3.86
CSI^‡^5 - Accepting Responsibility	4.27	2.64
CSI^‡^6 - Escape-avoidance	9.18	4.79
CSI^‡^7 - Problem Solving	8.97	3.87
CSI^‡^8 - Positive Reappraisal	9.80	4.76

* Mean;

† Standard Deviation;

‡ Coping Strategy Inventory

Some group differences in symptoms and coping strategies were found (Table 4). The effect sizes of the differences between the groups ranged from low to average. The analysis by gender, comparing the depression, anxiety and stress symptoms and the coping strategies employed, indicate statistically significant differences, with the female gender having higher scores in stress symptoms (U = 8,278.500, z =-2.229, *p* = 0.03) with small effect size (r =-0.12) and in Factor 6 - Escape-avoidance (U = 7,632.000, z=-3.110, *p* < 0.01,) than male graduate students, with small effect size (r =-0.17).

There were statistically significant differences between the graduate students who have a job and those who do not work, with the former presenting lower scores in depression symptoms (U = 9,748.000, z =-3.027, *p* < 0.01) with small effect size (r =-0.17) and higher scores in the following coping strategies: Factor 4 - Social Support (U=9,471.500, z =-3.374, *p* < 0.01), Factor7 - Problem Solving (U = 9,569.500, z=-3.252, *p* < 0.01) and Factor 8 - Positive Reappraisal (U = 10,432.500, z =-2.17, *p* =0.03) than those who do not work, with small effect size (-0.12 to-0.19). On the other hand, graduate students who do not work obtained a higher score in factor 6-Escape-avoidance (U = 10,602.500, z =-1.959, *p* = 0.05) with small effect size (r=-0.11) when compared to those who work. There were statistically significant differences in stress symptoms between graduate students who do not need to go out of their house to work and those who do need to do so, with the former having higher stress symptoms than the latter (U = 6,009.500, z= - 1.991, *p* = 0.05), with small effect size (r =-0.11).

The graduate students who practice some type of religion had lower scores in depression (U = 9,912.000, z =-4.354, *p* < 0.01, r =-0.24) and stress (U=11,823.500, z=-2.152, *p* = 0.03, r =-0.12) symptoms, and higher scores in the following coping strategies: Factor 2 - Distancing (U = 11,056.500, z =-3.047, *p* < 0.01, r =-0.17), Factor7 - Problem Solving (U = 1,073.000, z =-3.415, *p* < 0.01, r =-0.19) and Factor8-Positive Reappraisal (U = 7,669.500, z =-6.934, *p* < 0.01, r =-0.38) than those who do not profess any religion, with a low to near average effect (-0.12 to-0.38) However, those who do not practice any religion obtained a higher score in Factor6-Escape-avoidance (U=11,770.500, z =-2.215, *p* = 0.03) with small effect size (r=-0.12).

There were statistically significant differences in the depression scores (H(2) = 13.67, *p* =0.001) among students by study situation, with the group that “does not continue studying” having higher depression scores than those that “continue studying” (z=-3.073, *p* = 0.006, r = 0.17) and those who “study part-time” (z =-2.555, *p*=0.05, r=0.14). Regarding the differences in relation to the coping strategies employed, there were statistically significant differences in Factor 7 - Problem Solving, (H(2) = 12.96, *p* =0.02) with a higher score in the group of graduates who “continue studying” than in those who “do not continue studying” (z = 3.112, *p*=0.002), with small size effect (r=0.17).

There were statistically significant differences by type of institution in which the graduates study, in the depression scores (H(2) = 8.37, *p* = 0.02) between the public and private HEI groups (z = 2.882, *p* = 0.004), with the former presenting the highest score in these symptoms, with small effect size (r = 0.16). In addition, there were statistically significant differences in the anxiety symptoms scores (H(2) = 6.97, *p* =0.03) between graduate students from public and private institutions, the former with higher scores than the latter (z = 2.638, p=0.008) and with small effect size (r=0.14). There were no statistically significant differences across groups from public, private, and community HEIs concerning the coping strategies employed (*p* >0.05).

Analyses were performed to compare depression, anxiety and stress symptoms and the coping strategies employed by type of social distancing (total, partial, and none), with differences for the groups (H(2) = 17.956, *p* < 0.001). There were differences in the depression symptoms between graduate students who are in total social distancing when compared to those who are in partial social distancing with small effect size (z =-3.924, *p* < 0.001, r = 0.22) and between the graduate students who are in total social distancing when compared to those who are not in social distancing, with small effect size (z=-3.161, *p* = 0.002, r =-0.17). Regarding the anxiety symptoms, the group of graduate students who are in total social distancing presents higher scores than those who are in partial social distancing, both in anxiety (H(2) = 7.98, *p* = 0.02) and stress (H(2)=10.49, *p* = 0.005), with small effect sizes (z =-2.589, *p* = 0.010, r =-0.14) and (z=-3.063, *p* = 0.002, r =-0.17), respectively.

Regarding the coping strategies employed, there were no statistically significant differences between the groups by type of social distancing (*p* > 0.05). Spearman’s correlation analyses were performed to investigate the relation between the constructs under study. In general, there were significant correlations ranging from weak to moderate, with the highest correlations being found between Factor 6 - Escape-avoidance coping strategy and depression (rho = 0.60, *p* < 0.001), anxiety (rho = 0.53, *p* <0.01) and stress (rho = 0.64, *p* < 0.01) symptoms.

**Table 4:** Correlations of depression, anxiety and stress symptoms with coping strategies. Brazil

	Depression	Anxiety	Stress	CSI1*	CSI2^†^	CSI3^‡^	CSI4^§^	CSI5^||^	CSI6^¶^	CSI7**	CSI8^††^
Depression	1	-	-	-	-	-	-	-	-	-	-
Anxiety	0.70^‡‡^	1	-	-	-	-	-	-	-	-	-
Stress	0.76^‡‡^	0.79^‡‡^	1	-	-	-	-	-	-	-	-
CSI1*	0.18^‡‡^	0.27^‡‡^	0.36^‡‡^	1	-	-	-	-	-	-	-
CSI2^†^	0.04	0.13^§§^	0.06	0.20^‡‡^	1	-	-	-	-	-	-
CSI3^‡^	0.25^‡‡^	0.32^‡‡^	0.32^‡‡^	0.40^‡‡^	0.37^‡‡^	1	-	-	-	-	-
CSI4^§^	0.05	0.22^‡‡^	0.30^‡‡^	0.48^‡‡^	0.11^§§^	0.27^‡‡^	1	-	-	-	-
CSI5^||^	0.33^‡‡^	0.33^‡‡^	0.46^‡‡^	0.56^‡‡^	0.21^‡‡^	0.44^‡‡^	0.42^‡‡^	1	-	-	-
CSI6^¶^	0.60^‡‡^	0.53^‡‡^	0.64^‡‡^	0.40^‡‡^	0.12^§§^	0.36^‡‡^	0.21^‡‡^	0.45^‡‡^	1	-	-
CSI7**	-0.33^‡‡^	-0.08	-0.14^§§^	0.34^‡‡^	0.34^‡‡^	0.27^‡‡^	0.37^‡‡^	0.18^‡‡^	-0.16^‡‡^	1	-
CSI8^††^	-0.20^‡‡^	0.09	0.01	0.33^‡‡^	0.33^‡‡^	0.26^‡‡^	0.42^‡‡^	0.25^‡‡^	-0.02	0.61^‡‡^	1

* Coping Strategy Inventory 1: Confrontation;

† Coping Strategy Inventory 2: Distancing;

‡ Coping Strategy Inventory 3: Self-control;

§ Coping Strategy Inventory 4: Social Support;

|| Coping Strategy Inventory 5: Accepting Responsibility;

¶ Coping Strategy Inventory 6: Escape-avoidance;

** Coping Strategy Inventory 7: Problem Solving;

†† Coping Strategy Inventory 8: Positive Reappraisal;

‡‡ p≤0.01;

§§ p≤0.05

## Discussion

The first aspect that can be considered from the results is the difference between graduate students who hold jobs and those who do not, from a perspective that points out that those who work have fewer stress symptoms, greater social support, and a higher problem solving level. It is possible to understand this result considering that employment can be a factor that reduces anxiety in the face of the changes imposed by the pandemic^(17)^, especially in this group.

Instability in the face of the pandemic^(18)^ can trigger great emotional distress derived from practical aspects; for example, the possibility of resuming data collections and interventional studies, which were suspended by the pandemic. Therefore, employment could reduce stress precisely because it is a concrete dimension of employability and a context of greater stability than that represented exclusively by graduate studies. Considering the importance of social interactions in this context, the possibility of maintaining some social contacts at work also suggests greater social support compared to the more strictly isolated graduate student^(6)^.

In a scenario of instability in graduate education, the pandemic can accentuate some practical aspects that already concerned the students. Aspects such as maintaining scholarships or not, the deadlines for credit payments, the possible extension of deadlines, and even the opportunities of future employment, tend to impact on mental health. The pandemic has affected higher education. Situations such as layoffs, suspension of public tenders, and even availability of resources to maintain investments in graduate education indicate an uncertain future for those who aspire to an academic career^(6)^. This scenario can be more stressful for a student who is not yet employed. As observed in the sample, the response to this scenario is revealed in the greater use of escape-avoidance strategies, expanding the distance between the graduate student and the job market. In the context of the pandemic, these uncertainties also affect the possibility of continuing and even concluding graduate studies. In fact, one of the important aspects of adapting to the postgraduate context is financial security^(19)^. Thus, in this sample, maintenance of employment seems to have worked as a protective element against the symptoms of mental illness. The same can be said concerning continuity of studies. Together, these results may indicate that maintaining a work and study routine seems to be important for mental health in the population under study.

Some findings of this study are consistent with the well-established literature in the area, such as the relation of stress, depression and anxiety with the escape-avoidance strategy^(20-21)^. Potentially stressful and anxiogenic situations can trigger escape-avoidance in response to these symptoms. Another fact that corroborates the scientific literature refers to the greater exposure of women to stress symptoms^(22)^. This discussion cannot be separated from a gender perspective that considers the role of women in our society. In the context of social distancing, women tend to assume different activities more intensely.

In the case of female graduate students, these activities can very probably involve not only continuing education but also caring for the home, children, and other family members, in a situation aggravated by the pandemic. This could also explain that these women tend to use escape-avoidance strategies more intensely, in a context that does not provide conditions to balance dedication to the various activities demanded during the pandemic, with the collaboration of other family members around them. This should also be considered since students who are isolated at their homes tend to manifest more stress symptoms.

Concerning the coping strategies employed, there was greater positive reappraisal, followed by a lesser level in accepting responsibility. Regarding this last factor, it can be understood that the situation of isolation is an important marker in this context. Concerning household activities, the “accepting responsibility” strategy can be related to the division of tasks and to the very effect of greater proximity to family members who live in the same house.

Regarding graduate studies, the instability introduced by the pandemic interrupted normal academic processes such as in-person data collection, as well as it postponed qualification and defense exam dates, needing adjustments in schedules and deadlines. As the pandemic is an external event, a global marker, it is proposed that these graduate students would have a lesser sense of control, which would result in a lesser sense of responsibility in the face of the changes that affect and will still affect them. The possibility of facing these issues and conducting deeper internal reflections could explain a greater positive reappraisal. This aspect can also be considered protective when reinterpreting the conditions imposed by the pandemic and accessing more adaptive responses to the challenges triggered during this period.

A significant element in this study is religiousness/spirituality. Although the research has focused on professing a religion, it is noteworthy that the students who are religious have lower stress levels, which is supported by the scientific literature^(23-24)^. Religious belonging as a form of contact with a broader dimension and connection with something superior or external to reality can be associated with a greater degree of distancing, presented in this study as a coping strategy^(24)^. In this context, distancing from reality would not be equivalent to an escape from concrete elements and repercussions of this pandemic, but a protective resource that allows for reflections and, most probably, problem solving. Thus, the religiousness of this sample is associated with a more protective behavior in terms of mental health. As previously discussed, a similar analysis can be extended to the employment context.

The results of this study must be appreciated in the face of some limitations. The small effect sizes found in different scores between the variables require the results to be interpreted with caution. In addition, it is important to consider the potential biases inherent to the design used, mainly with regards to the electronic cross-sectional survey. However, this design has been increasingly employed, especially considering the need to conduct research studies on a remote basis in the pandemic context. The sociodemographic characteristics of the sample should be considered sparingly, such as the prevalence of the female gender, the majority of students being from the South-Southeast axis of the country, white-skinned and from graduate programs offered by public universities. Although this profile can portray graduate studies in the country, it must be considered that it can make the population more directly affected by the pandemic unfeasible and which, for example, can be at greater vulnerability, incurring in the possibility of abandonment or even of not concluding their graduate studies.

The debate proposed must consider educational policies devised in the face of the COVID-19 pandemic in Brazil. In a precarious educational context, with difficulties in responding to the challenges imposed by the pandemic, the adaptation of graduate education to this context seems to be a distant element in this discussion. Thus, it is suggested that, up to date, this is the first study focusing on the mental health and coping strategies of graduate students during the COVID-19 pandemic. It is argued that the reflection on the mental health of graduate students cannot be constructed without contextual elements such as the precariousness of teaching and the pandemic being also brought up.

In practical terms, the results obtained indicate the need for investment in intervention strategies for graduate students that take into account two elements - one strongly linked to individuality, such as religiousness/spirituality, and the other of a collective nature: employment. Such elements were configured as potential strategies associated to the improvement of mental health in this population. Therefore, it can be an important point at this moment of greater instability to allow the category of religiousness/spirituality to be better discussed in health and training contexts. Likewise, employment of graduate students must be considered beyond a mere protective element. It should be thought of as a necessity in their studies and not only as an internship which possibly prevents these students from contributing to society with their research.

From the panorama discussed in this study, the highlights are summarized as follows: focus on possible coping strategies produced in response to COVID-19; maintaining a study routine is correlated with lower scores of mental disorders; religious practice is correlated with lower scores of mental disorders; entering the labor market and religiousness should be protective factors; and recognition of the psychological needs of graduate students in the pandemic.

## Conclusion

Our findings pointed out that maintaining a work and study routine, as well as religiousness, can act as protective factors for mental health during the pandemic. Holding a job can be related to the perception of greater material security, as well as providing contact with people who serve as social support, the most frequent coping strategy in graduate students who work. Religiousness can act as a protective factor by providing resources for the individual to distance from the problem and to reevaluate it in a protective manner. One of the most frequently given pieces of advice to mitigate the impact of the pandemic on mental health is not to consume too much current information, looking for activities that can help “disconnect” for a while. Thus, religious practice can provide moments of withdrawal from the problem, allowing it to be reframed, in some way.
